# How the stigma of low literacy can impair patient-professional spoken interactions and affect health: insights from a qualitative investigation

**DOI:** 10.1186/1472-6963-13-319

**Published:** 2013-08-16

**Authors:** Phyllis Easton, Vikki A Entwistle, Brian Williams

**Affiliations:** 1 NHS Tayside, Kings Cross, Clepington Road, Dundee, DD3 8EA, UK; 2 University of Aberdeen, Health Services Research Unit, Foresterhill, Aberdeen, AB25 2ZD, UK; 3 University of Stirling, Stirling, FK9 4LA, UK

**Keywords:** Low literacy, Patient-provider communication, Patient-provider relationships, Person-centred care, Qualitative

## Abstract

**Background:**

Low literacy is a significant problem across the developed world. A considerable body of research has reported associations between low literacy and less appropriate access to healthcare services, lower likelihood of self-managing health conditions well, and poorer health outcomes. There is a need to explore the previously neglected perspectives of people with low literacy to help explain how low literacy can lead to poor health, and to consider how to improve the ability of health services to meet their needs.

**Methods:**

Two stage qualitative study. In-depth individual interviews followed by focus groups to confirm analysis and develop suggestions for service improvements. A purposive sample of 29 adults with English as their first language who had sought help with literacy was recruited from an Adult Learning Centre in the UK.

**Results:**

Over and above the well-documented difficulties that people with low literacy can have with the written information and complex explanations and instructions they encounter as they use health services, the stigma of low literacy had significant negative implications for participants’ *spoken* interactions with healthcare professionals.

Participants described various difficulties in consultations, some of which had impacted negatively on their broader healthcare experiences and abilities to self-manage health conditions. Some communication difficulties were apparently perpetuated or exacerbated because participants limited their conversational engagement and used a variety of strategies to cover up their low literacy that could send misleading signals to health professionals.

Participants’ biographical narratives revealed that the ways in which they managed their low literacy in healthcare settings, as in other social contexts, stemmed from highly negative experiences with literacy-related stigma, usually from their schooldays onwards. They also suggest that literacy-related stigma can significantly undermine mental wellbeing by prompting self-exclusion from social participation and generating a persistent anxiety about revealing literacy difficulties.

**Conclusion:**

Low-literacy-related stigma can seriously impair people’s spoken interactions with health professionals and their potential to benefit from health services. As policies increasingly emphasise the need for patients’ participation, services need to simplify the literacy requirements of service use and health professionals need to offer non-judgemental (universal) literacy-sensitive support to promote positive healthcare experiences and outcomes.

## Background

Low literacy is a significant problem across the developed world. The OECD International Adult Literacy Survey reported 22% of adults in the USA and 23% of adults in the UK performed at the lowest level of a 5-point scale of functional literacy [1]. A recent UK study has highlighted the implications of low functional literacy for poor health literacy [2].

The definition of health literacy continues to evolve and remains the subject of much debate in the literature [3]. One definition that reflects the evolution towards broader relevance across health contexts is that health literacy is *“the wide range of skills and competencies that people develop to seek out, comprehend, evaluate and use health information and concepts to make informed choices, reduce health risks and increase quality of life”*[4].

Many of the measures of health literacy that are currently available assess only limited aspects of the concept [5]. They tend to focus on literacy skills, so people who are functionally literate can score high even though they may have low health literacy according to the definition given. People with low functional literacy are less likely to achieve high health literacy however it is measured, not least because of the heavy reliance on written information across healthcare services.

Low functional or health literacy is strongly correlated with poorer health outcomes and poorer use of health resources [6]. People with low functional or health literacy generally have less appropriate access to services; [7-9] including more hospitalisation, greater use of emergency services and lower uptake of preventive health services [10]. They have poorer knowledge and self-management of health conditions such as asthma, [11,12] and diabetes; [13,14] and poorer ability to take medicine properly [10]. They are less likely to exercise, more likely to smoke, more than twice as likely to report being in ‘poor’ or ‘very poor’ health [15] and have a higher risk of mortality [16].

A person’s low literacy may be obvious to others when it is associated with language difficulties or significant cognitive impairment. In clinical contexts, these cues may prompt some health professionals to offer support, including interpretation and simplified communication. However, several factors tend to create and maintain a population whose literacy difficulties remain hidden from healthcare professionals. These factors include individuals’ lack of recognition or acknowledgement of their own low literacy [15,17]; the shame and stigma associated with low literacy [18]; and low awareness among healthcare staff of potential difficulties with reading, writing or numeracy in the population of patients with no obvious language difficulties or cognitive impairments [19-21]. A recent review has confirmed that people whose low functional or health literacy may not be obvious to healthcare staff have poor health outcomes similar to those whose difficulties are more readily apparent [22]. These poor outcomes may be modifiable even though the contributing difficulties are hidden.

The causal pathways that have been postulated between low literacy and poor health have been based primarily on cross-sectional quantitative research. Paasche-Orlow and Wolf in their conceptual model of the causal pathways between limited health literacy and poorer health outcomes offered three domains: access and utilisation of healthcare; patient and provider interaction; and self-care. Within each of these postulated domains, they identified both patient factors and factors external to the patient as significant mediators of outcome [23]. Their conceptual model has been further developed by others, most notably in a later paper, which drew out motivational and volitional determinants from social cognition models. These included the ability of people with low literacy to obtain knowledge. However, the healthcare and self-management experiences of people with low literacy themselves have been largely neglected in research.

Experiential accounts are needed to help develop nuanced explanations of how low literacy can lead to poor health and to inform the development of initiatives to support people with low literacy to access and benefit from health services. The need for these accounts has been made more pressing by the promotion, in the UK and elsewhere, of policies and action plans that seek to address health inequalities and improve population health via asset based, co-production approaches involving patients as partners [24,25] and person-centred care which promotes *‘mutually beneficial partnerships between patients, their families and those delivering healthcare services which respect individual needs and values and which demonstrate compassion, continuity, clear communication and shared decision-making’*[26]. Successful delivery and comprehensive evaluation of these approaches is likely to require a robust understanding of the particular issues faced by people with low literacy and of the causal pathways between low literacy and poor health.

### Objectives

We report on a study that sought to:

1. Investigate and illuminate previously neglected pathways between low literacy and poor health by exploring the perspectives of people with low literacy on access to healthcare services, self-management of health conditions and health behaviours

2. Consider how health services might better support people with low literacy and reduce the tendency for them to experience disproportionately poor health.

## Methods

### Design

This qualitative study used individual face-to-face in-depth interviews to explore personal experiences of literacy, healthcare (including self-care) and health, then focus groups to check the resonance of the analysis of the individual interviews and to further explore ideas about how health services could improve support for people with low literacy. Interaction through qualitative interviewing offered a more appropriate approach than structured interviewing or the use of a questionnaire. Any type of data gathering which required the participants to engage in reading or writing activities had to be discounted. Face-to-face individual interviews had an advantage over focus groups in the first instance because they would be confidential, and would allow for greater in-depth exploration of individual perspectives. We acknowledged that participants’ experiences of health and healthcare may be sensitive and they may not wish to share these in a group. Focus groups were conducted to provide an opportunity to check our summary of findings (helping to establish the credibility of the scope and analysis of our data from individual interviews), and to discuss further the ideas about what health services could do that had been suggested by those participating in the initial interview process. The focus group topics were less personal or sensitive than those covered in individual interviews, and the discussion benefited from the group interaction.

### Setting

A community-based adult learning centre in Dundee, UK. Dundee is Scotland’s fourth largest city, with a population of 146,000. Dundee suffers high levels of deprivation, with approximately 30% of the population living in areas that are among the 15% most deprived in Scotland [27]. An estimated 6–9% of Dundee residents belong to a minority ethnic group [28].

### Participants and recruitment

Participants were adults of working age who had English as a first language and had sought help with literacy difficulties from adult learning services. We adopted these inclusion criteria because of our interest in the population with low literacy that is potentially hidden within health services. We did not administer a formal literacy assessment during or after recruitment because we wanted to avoid embarrassing potential participants, particularly while the assessment would bring them no particular benefit. Instead, participants were asked about what literacy support they were accessing at the beginning of the interviews. One author (PE) visited adult literacy classes and described the purpose of the research and the interview process. She provided simple participant information sheets and consent forms which literacy tutors worked through in class learning time to ensure understanding. Literacy tutors assigned interview times to people interested in participating or, with agreement, passed on telephone numbers to PE. Literacy tutors recruited focus group participants from their classes. Everyone who chose to take part signed a simple consent form.

Demographic information was collected during the interviews. Experience of particular health problems was not a prerequisite to participation: we recognised that most people will at some time attend their general practitioner, need to obtain over the counter or prescription medicines from a pharmacy, and consider how their behaviours might affect their health. Participants were asked about any health conditions that required self-management and/or regular contact with health services as well as experiences of generally taking care of their health. Participants were also asked their age at the end of the interview. Recruitment was then monitored to ensure the inclusion of men and women of different ages, varying levels of reading, writing and numeracy support needs; and with different experiences of health problems and healthcare, ranging from people who had long term conditions requiring regular self-management to people who were generally healthy and had experienced minor illnesses only. Each participant was given a £10 store voucher after their interview.

Ethics approval was granted by the University of Dundee Research Ethics Committee (UREC 8060).

### Data gathering

One author (PE) conducted the individual interviews in private rooms within the Adult Learning Centre. She used a topic guide (Table 1) to ensure coverage of key issues identified in the literature, but adopted a conversational style. Interviews opened with a warm up question about how the person had come to adult learning and what kinds of help they were accessing. This question also helped establish their stage of literacy education. Participants were then asked about their health and any ways in which they thought their literacy affected their health; their experiences of looking after their own or family members’ health (for example, how they got on with prescriptions); and their experiences of using health services. Our initial analysis of early interviews suggested that childhood experiences of being singled out, demeaned and bullied because of literacy difficulties could be significant, and this encouraged us to ask directly and in more detail in subsequent interviews about experiences at school and about disclosing literacy issues to others; these topics were added to the interview guide (Table 1).

**Table 1:** Interview topic guide

**Interview topic**	**Example questions**
Introductions and warm up	Can you start by telling me how you came to get help?
	Do you have anyone who regularly helps you with (literacy)*?
How general health may be affected by literacy level	How would you say your general health is?
	Can you tell me about of any ways your (literacy) affects your health?
Self care: health information and preventive behaviour	Where do you normally learn about things to do with health?
	Do you attend or take part in screening?
	- cervical (females only)
	- breast (females over 50) bowel (all over 50)
Self care: management of health problems; medication; family health	Do you or anyone you look after have a medical condition that needs to be treated or checked up on regularly?
	How do you get on with prescriptions?
	Do you have children? Tell me about what you have to do to look after their health–immunisation, childhood illnesses etc.
Access to health services: patient-healthcare provider relationships; navigating the health service environment	Does your GP know that you have/have had some difficulties with (literacy)?
	If yes, how did do they know?
	Does your (literacy) affect whether you go to health services? If yes, how?
	Do you have anyone who helps you when you use health services?
Types of initiatives that would help access to services/self-care etc.	What would make it easier for you to …… take care of your condition/take your medicine properly? (these will depend on the responses to previous questions)
	How could the ……….. service be improved for people with any literacy difficulties?
Additions to topic guide after initial analyses (questions added to ensure coverage of these emerging topics)	
Life history	How did you get on at school?
	When were you aware you had needed some help with your literacy?
	What sort of work have you done in the past?
Disclosure management	Who knows about your literacy?
	What did you say to them when you told them?
	How would you decide who to tell?
Advantages/disadvantages of disclosure	Do you think it matters if healthcare staff know about your literacy?

*The word ‘literacy’ was replaced in each case by the terms used by the individual in describing their need for literacy support.

We continued to analyse completed interviews concurrently with data gathering and agreed that no substantial new issues were appearing when 25 interviews had been completed. The characteristics of the 25 participants are summarised in Table 2.

**Table 2:** Characteristics of participants

**Characteristic**	**Individual interviews**	**Focus groups**
	**(N?=?25)**	**(N?=?9)**
**Gender**		
Female	18 (72%)	6 (67%)
**Age band**		
Teens	4 (16%)	1 (11%)
20s	6 (24%)	1 (11%)
30s	3 (12%)	1 (11%)
40s	5 (20%)	3 (33%
50s	7 (28%)	3 (33%)
**Employment**		
In paid employment	8 (32%	Not recorded
Not in paid employment	14 (56%)	Not recorded
Training for employment	3 (12%)	Not recorded
Long term condition*		
Yes	14 (56%)	Not recorded
**Participation**		
Previously interviewed	Not applicable	5 (55%)
New to study	Not applicable	4 (44%)

*Participants were considered to have a long term condition if, on questioning, they reported any health problem that lasts a year or longer, impacts on a person’s life, and may require ongoing care and support.

PE undertook two focus group discussions within a classroom in the Adult Learning Centre. They were conducted after a preliminary analysis of the individual interviews and used to help confirm the appropriateness of analytic themes, and to allow further discussion of ideas about what health services could do. PE summarised the main findings one theme at a time, using a topic guide as a reminder (Table 3), and encouraged discussion of each theme. The themes were introduced as topics that had been mentioned by some or all of the individual interviewees. After comments and discussion, if the perspectives or issues that had arisen in individual interviews were not expressed, these were shared and further comments and discussion invited.

**Table 3:** Focus group topic guide

**Topic**	**Issues raised by individual interviewees**
Access to health services	Appointment letters; hospital signs
Relationships with healthcare staff	Language used
Disclosure to healthcare staff	Who to tell; how to tell
Self-management of health conditions	Obtaining and using medicines
Stigma and mental wellbeing	Other people’s attitudes; disclosure management
Suggestions for the NHS to improve the experiences of people with low literacy	Colour coding; oral explanations

The focus groups involved 5 people who had previously been interviewed and 4 new participants (Table 2). Employment and health data were not collected from focus group participants.

### Analysis

Interviews and focus group discussions were audio-recorded and transcribed verbatim. Framework [29] was the preferred method of analysis because it follows a well-defined procedure and provides a visible method in which data and analytic ideas can be viewed, reconsidered and reworked. In accordance with Framework Analysis, printed transcripts were read and an initial list of themes and concepts drawn up. From these, we developed a coding framework (Table 4) which was applied systematically to the interview transcripts. Code labels were entered manually in the margins of the printed transcripts. Complete transcripts were retained and revisited as new themes emerged, and the coding framework was developed and revised.

**Table 4:** Coding framework: key themes and concepts from participant interviews

**Theme**	**Sub-themes**
**1. Personal details**	1.1 Age group
	1.2 Gender
	1.3 Household arrangements
	1.4 Employment status
	1.5 Length of time at centre
	1.6 Reported causal factors associated with literacy
	1.7 Literacy learning needs
	1.8 Reasons for seeking help with literacy
	1.9 Health status
**2. Literacy-related life history**	2.1 Education
	2.2 Employment
**3. Communication and relationships with healthcare staff**	3.1 Written communication
	3.2 Spoken communication
	3.3 Relationships with GPs
	3.4 Relationships with other healthcare staff
	3.5 Other issues
**4. Disclosure of literacy difficulties**	4.1 Disclosure to healthcare staff
	4.2 Disclosure to others
	4.3 Selective disclosure
	4.4 Strategies to avoid disclosure
	4.5 Other issues
**5. Coping strategies to access services and carry out self-care activities**	5.1 Asking for help
	5.2 The role of others
	5.3 Coping devices
	5.4 Other
**6. Health literacy**	6.1 Accessing and navigating health services
	6.2 Obtaining and using health information
	6.3 Knowledge and understanding of health conditions and treatment
	6.4 Other issues
**7. Mental wellbeing**	7.1 General mental wellbeing
	7.2 Problem attribution
	7.3 Stigma
	7.4 Stress
	7.5 Confidence
	7.6 Social experiences
	7.7 Other
**8. Suggested changes to health service**	8.1 Written communication
	8.2 Spoken communication
	8.3 Hospital environment
	8.4 Awareness and acknowledgement of low literacy
	8.5 Facilitating disclosure of low literacy
	8.6 Avoiding disclosure of low literacy
	8.7 Other issues

Once transcripts had been labelled, key data and points of interpretation were summarised in charts, which we produced manually in Microsoft Excel. We entered one participant per row and one sub-theme per column to allow for vertical (across the participant sample) and horizontal (within individual participants) analysis. The three authors met regularly to discuss and agree definitions of codes and the interpretation of the data. Illustrative quotations are provided in the findings section. All participant names have been changed.

## Results

Most participants reported accessing support for a range of literacy learning needs including reading, writing and numeracy. All clearly faced significant challenges with literacy activities, although their particular skills and challenges varied. For example, some people struggled to read simple material while others could read at a higher level but had been prompted to seek support with writing because new practices at work or a need to change jobs meant writing skills were needed. Some people said they were attending the learning centre to improve their existing skills, including their ‘English’ (although all were native speakers). Several revealed, either explicitly or indirectly during their interviews, that their understanding of what words and text they could read was poor.

### Difficulties with written communication

Not surprisingly, participants gave various examples of difficulties they had had with written communication in healthcare contexts. The heavy reliance of the health service on written text, and the widespread use of medical terminology and jargon, had variously led people to miss or be late for appointments, to arrive unprepared for planned interventions, to struggle with leaflets or forms handed out ‘on the spot,’ to fail to follow instructions or requests, and (compounded by any of these) to feel anxious and stressed before and during any conversations with clinicians.

“*I’ve been to the hospital a few times and they’ve been like ‘Oh you were meant to bring a urine sample’ and I was ‘Oh I didn’t know’......... cos I just read the date, the time and the ward”* (Karen, female, 20s)

*“I could read the word ‘endoscopy’ .... I actually thought an endoscopy was down here* [indicating throat] *but I was told by the doctor that they’re checking your stomach, see so I didn’t think they were going the other way, so it was quite ‘bloody hell,’ you know and you don’t realise what you’re gonna feel like after so it was quite an ordeal*.” (Jack, male, 40s)

Our findings in relation to written communication are in line with previous research [30-35].

### Difficulties with spoken communication

Our two main original findings are (1) that, over and above the well-documented difficulties that people with low literacy can have with the written information and complex explanations and instructions they encounter as they use health services, people with low literacy can have significant difficulties in their *spoken* consultations with health professionals and (2) that one key to understanding their difficulties in spoken conversations lies in recognising an often strong reluctance to ‘disclose’ difficulties with reading, writing or understanding in social and healthcare contexts that is rooted in the terrible stigma that can be associated with low literacy.

Participants’ accounts revealed a range of difficulties that they experienced with spoken communication in health care contexts. These difficulties impacted negatively on participants’ experiences of formal healthcare and abilities to manage their health conditions at home. Some of the difficulties were apparently perpetuated or exacerbated because participants limited their conversational engagements with health professionals and often took care to avoid revealing when they did not understand what was being discussed.

Participants frequently reported difficulties in understanding what was said during consultations and they attributed these difficulties in part to the language clinical staff (especially doctors) used. Participants’ descriptions of spoken conversations included terms such as, “*gobbledygook;” “big fancy words;”* and *“twenty four letter words.”*

Some participants thought some doctors made no effort to help them understand what was being said. For example:

*“.... and he* [hospital consultant] *was ‘blah blah blah’ and he knew fine I didn’t have the foggiest idea what he was talking about.”* (Harry, male, 40s)

Some participants clearly recognised that their own anxieties could also contribute to their difficulties with understanding what was said in consultations, both because they impaired listening and concentration, and because they deterred them from asking questions. For example, Louise described how she felt every time a healthcare professional produced a piece of paper:

*“… I’m like that, ‘Oh no, they’re wanting me to write something,’ start panicking and that seems to take over you and sometimes you’re like that, ‘What was they saying there?’ because the anxiety’s took over what’s going on.”* (Louise, female, 40s)

It was particularly striking that, in spite of their self-recognised difficulties in grasping potentially important information, a number of participants described feigning understanding to the health professionals they were talking with:

*“…they never explain anything properly. It’s always their own big words and I just say, ‘Yeah, okay’ and I go home and I’m like, ‘I don’t know what that meant.*’” (Megan, female, teens)

The recurring reason given by participants who said they were reluctant to ask questions or to make it known that they had not understood what was said was that they were concerned to avoid revealing to health professionals that they had literacy difficulties.

### Engagement limited by fear of disclosure of low literacy

The concern to keep their literacy problems hidden from health professionals was explicitly shared by most participants in their interviews, and all mentioned some occasions on which they had concealed their low literacy in healthcare settings, for example, by: saying they didn’t have their reading glasses; relying on a trusted person who accompanied them to consultations; saying they would read something later; or making an excuse to leave.

Some participants seemed almost wracked with fear that health professionals might discover their low literacy, and regularly limited their conversations with health services as a result. For example, Barbara described what happened when she was asked to complete a form at a first appointment and said she had done this kind of thing many times:

*“..... I couldn’t spell it. I just went, ‘You know what, I’m going to have to go. I’m not feeling very good. I’ll come back, I will come back’ and I grabbed it* [the form] *and ran out.”* (Barbara, female, 50s)

Barbara also described what she felt like and did within consultations, especially if she saw paperwork:

*“…. you’re nervous and you’re pulling back ...... then you’re just going to finish it as quick as you can, short answers, just get out. ‘I don’t know’, or ‘Yeah’, ‘No’, where you wouldn’t say, ‘Well, actually.....’ and be more explicit, you wouldn’t do that. Well, I wouldn’t. I’d want out.”* (Barbara, female, 50s)

Not all participants were so strongly concerned to conceal their low literacy, and a minority of our sample told us that they would reveal their low literacy to a healthcare professional if they were asked directly and if they felt comfortable with the person who was asking. However, few were able to recount explicitly having revealed their difficulties to any healthcare staff.

When asked what they thought *would* happen if their literacy became apparent to healthcare staff, participants talked primarily in terms of possibly being looked down on, or being suspected of having been somehow irresponsible in respect of their own or their child’s health. For example:

*“They* [healthcare staff] *could treat you differently, and not in a nice way. They could look upon you as if you’re stupid, you don’t understand what I’m telling you.”* (Karen, female, 20s)

*“… when something bad does happen or there’s an accident or something when you first go to the doctor or the hospital …you’re sitting there thinking like ‘Do I tell them do I not?’ but you’re scared to in case then they twist and they think ‘Well this could have happened because you’ve done wrong.’”* (Katy, female, 20s)

Despite these fears of what *might* happen, none of our participants could recall any *actual* experience of having been treated badly in practice as a result of healthcare professionals finding out about their literacy difficulties (most had been careful to avoid healthcare professionals finding out). Some had, however, experienced poor interactions when they had sought ways around the use of written text. For example, a young mother described the response when she asked a midwife for help with bathing her newborn baby:

*“… you’re saying …‘Could you show me … cos I’m not too sure’ …. and she went ‘Oh (sigh) I just gave you a leaflet.’”* (Katy, female, 20s)

Some participants were aware that the behaviours that they used to conceal their low literacy (as well as any literacy-related mistakes they might make) could be misinterpreted:

*“….if an appointment came…..instead of filing it or writing it down in the diary I’d just put it away and just forget … people were getting a bit like ‘What’s she playing at?’”* (Dorothy, female, 50s)

### Low literacy and social-psychological wellbeing

Participants’ reluctance to reveal their low literacy to health professionals was consistent with their broader reluctance to reveal these difficulties to anyone (other than select family and friends). Overall, participants identified a range of strategies that they used to cover up their literacy difficulties. We heard, for example, of people pretending to read newspapers during work breaks, leaving training sessions at times when there was a possibility of being asked to read or write, avoiding participation in a range of social activities, and even colluding with peers’ expressions of negative attitudes about people with literacy difficulties:

*“I never told anybody, it was always just something that was hidden away back so nobody could see it. I don’t know why. I think that’s just no confidence, not wanting to tell anybody in case they think I’m an alien.”* (Megan, female, teens)

*“I think I’m doing what most people do that can’t read you step back from the crowd. Especially in a group or something because the worst thing that could ever happen is ‘oh (name) could you just read that line for me?’ Boof, your whole world just sinks, you know what I mean. There’s nothing worse and that’s why people always step back near the door just in case that happens.”* (Bert, male, 40s)

*“… and they were saying* … ‘*Oh, there was people at* [a named group] *and they can’t even read and write’ and I’m just sitting there going ‘oooh!’ Things like that. I wouldn’t like my own friends to know*” (Louise, female, 40s)

As one focus group participant summed up, they felt they were always *“having to be one step ahead in case you’re handed a form or something.”* (Jason, Focus Group 1)

Some participants thought that both low literacy and its associated stigma had impacted on their health in ways not mediated by their difficulties with healthcare use or the management of health conditions at home. In particular, literacy-related stigma was seen as a negative influence on mental wellbeing. For example, Harry talked extensively about how the stigma of low literacy, the self-imposed exclusion from social activities and the perpetual concern to avoid disclosure *“wears you down.”* He quite clearly thought that his literacy problems had contributed to him feeling socially isolated and *“weak and depressed.”*

Several other participants volunteered that they had been diagnosed with stress and other mental health problems including nervous breakdown, depression, eating disorder and panic attacks. There was nothing in their accounts to suggest that the diagnosing doctors had uncovered or explored low literacy as a possible contributing factor.

### Reluctance to disclose low literacy rooted in early experiences of stigma

Our early analyses of participants’ narratives suggested that early experiences of being labelled, stigmatised and discriminated against had been strongly influential in people’s behaviours in healthcare contexts and other social situations. This tentative theory was confirmed in subsequent interviews and during the two focus group discussions.

It became evident from participants’ biographical narratives that the literacy-related fears that impaired their social engagement could usually be traced back to childhood experiences. Often without any specific prompting, participants offered information about their schooldays in their interviews. They recounted struggles both with literacy and with being labelled and discriminated against because of these. They reported being humiliated by teachers and picked on and bullied by other children. For example:

*“… my teacher thought making me read out to the school was one of the best things there was. It wasn’t. It was not. It was so… ..the children, they were the worst cos it was like another notch on… Oh (sighs).. ‘Well, she’s thick, stupid, now she can’t talk.’”* (Margaret, female, 50s)

These kinds of experiences and subsequent avoidance and cover-up behaviours recurred throughout participants’ life stories. The challenges they faced and the sometimes dysfunctional ways they reported behaving in healthcare contexts, such as limiting conversations with healthcare staff or covering up their lack of understanding to avoid anticipated stigmatisation was explicitly recognised by some as being directly linked to their childhood experiences:

*“I’m no asking questions cos at school when you ask … kids that ask questions and don’t know what they’re askin’ they get humiliated and humiliated and it goes on and on and on and then it suddenly becomes part of your make up*…” (Harry, male, 40s)

### Participants’ suggestions for improvement in the health service

Some participants clearly believed that healthcare staff “*think that everyone can read”* and suggested a need to educate them about dyslexia and literacy problems. We also heard a variety of other suggestions for service improvement.

Not surprisingly, participants advocated the simplification of written information, including signage, appointment letters and instructions for medicine-taking, as well as healthcare leaflets. They also suggested that their understanding of clinical information and advice could be much improved if healthcare professionals explained things in lay terms rather than using medical terminology and jargon.

For the minority of participants who indicated that they *would* reveal their low literacy to healthcare professionals they felt they could trust, attempts by health services and health professionals to make it easy for people to ask for help with literacy activities without being judged seemed potentially useful–although they still did not like the idea that they might need to tell every health professional they met. Most participants, however, strongly preferred to avoid disclosing literacy difficulties and some suggested that their resolve to conceal their low literacy would be further strengthened if they were asked to identify themselves as needing help. These people favoured what are sometimes called ‘universal’ solutions, for example, simplifying written information, jargon-free simple explanations and advice or providing recordings of key consultations–for everyone, rather than as an option for those who are seen to require it [36]. Participants also suggested that if services sent forms out before appointments, they could avoid putting people on the spot; that departments and relevant signposts within healthcare facilities could be colour coded; and that technology could now be used to provide interactive audio and visual information and instructions.

## Discussion

This study is among the first to elicit first-hand accounts of experiences with healthcare from people with low literacy living in the UK. We believe it is the first to focus on the ‘hidden population’ whose low literacy may not be obvious to healthcare staff and others. The study has illuminated previously unrecognised sources and implications of the stigma associated with low literacy, and has highlighted the difficulties that people can have with spoken as well as written communication in healthcare contexts. Most notably, we have presented findings that extend understanding of the ways that literacy-related stigma can undermine spoken interactions and relationships between patients and healthcare staff (important aspects of patients’ experiences of the quality of healthcare delivery), [37] impair patients’ engagement with healthcare provision, and limit their abilities to self-manage their health conditions at home. We have also highlighted the potential negative implications of literacy-related stigma for people’s mental wellbeing–which could perhaps be seen as an example of a ‘direct’ (i.e. not just service-mediated) impact of low literacy on health.

The stigma associated with low literacy has been acknowledged previously in health-related research: Parikh et al. reported that of 58 patients with low literacy, two thirds (67.2%) indicated in response to structured survey questions that they had not told their spouses and 19% that they had told no one [18]. Our study has now helped to explain *how* literacy-related stigma can feature significantly in the causal pathways that link low literacy to poor health, for example, by prompting ‘cover-up’ behaviours that impede engagement with healthcare staff and so threaten the integrity of healthcare consultations, and by limiting information acquisition and help-seeking and so hampering self-management activities. Our study also tends to confirm that the kinds of literacy-related concerns identified by studies conducted in the USA can occur in the UK too. For example, participants highlighted the many aspects of health service use that currently depend on written communication, and the inaccessibility of these to people with low literacy. This has been recognised previously [30-33]. They expressed concern that health service staff were often unaware, or underestimated the difficulties that people might have with reading and writing–a concern that studies of healthcare professionals from the USA suggest is likely to be justified [19,20].

### Strengths and weaknesses of the study

One-to-one semi-structured interviewing generated data and insights that would not have been obtained by more structured, closed methods. In particular, by listening to personal biographical narratives we were able to identify explanations for participants’ guarded relationships with healthcare staff and their continued concealment of their low literacy. The focus groups allowed some of those interviewed previously and some new participants to comment on our interpretation of key findings and to discuss proposed service responses in more detail. Data were analysed using a recognised systematic approach.

By recruiting participants via adult learning classes, we potentially limited the findings to people who were prepared to be open about their literacy issues–at least in some contexts. It would have been difficult to identify people who had not recognised or sought help for their low literacy in an ethically acceptable manner for research purposes only. Although participants in this study may have been more willing and able than some to acknowledge and address their literacy issues, their willingness and confidence appeared to be restricted to particular contexts such as accessing adult learning. There was no evidence in our data that their having revealed their low literacy in this particular context significantly influenced their disclosure behaviours in other contexts. Consistent with our interest in the ‘hidden population’, few participants had disclosed or sought help with their low literacy within health services.

We achieved a diverse sample, although there were more women than men within it. We included some participants whose literacy difficulties had recently lessened to some extent as a result of their participation in adult learning. These people were able to talk about some issues relating to their low literacy that they had not recognised until they started attending the Adult Learning Centre and acquired a new perspective on them.

### Implications for policy and practice

Given the prevalence of low literacy, most frontline clinical staff in many developed countries will encounter people with literacy difficulties every day. This study highlights several implications for both health service policy and health professionals’ practice. In general terms, the study findings (including those confirming the difficulties people have with written communication, which we have not reported in detail here) lend support from patients’ perspectives for calls to make changes to healthcare systems and to the health information that is offered by health professionals, in order to make them more engaging for people with low literacy [38]. This will be particularly important for the implementation of the various health service policies evident in the UK and elsewhere that seek to ensure that healthcare is person-centred and that patients are supported as active partners in decision-making about their own care and in the management of their long-term conditions [24,26,39]. In practice, opportunities for people with low literacy to work in effective partnerships with staff will be substantially diminished by the kinds of problems our study has identified unless communication and relationships improve. A reliance on information provision to activate patients and facilitate a shift in decision-making and care-provision responsibilities risks exacerbating social inequalities in access to good quality care and outcomes: in the absence of supportive interpersonal relationships with staff, people with literacy difficulties are particularly likely to struggle [40].

In terms of the planning and provision of support for self-management, our study has highlighted a need to review and provide alternatives to peer-support activities, and especially group based training for people living with long-term conditions. Given the concerns about stigma and the avoidance strategies that we heard about from our study participants, group-based education is unlikely to be an attractive or effective option for people who struggle with literacy-related activities. To complement efforts to reduce the literacy demands that health service systems and processes make on health service users, interventions are also needed to raise professional awareness that low literacy is fairly common and potentially has a range of negative consequences for people’s willingness and ability to engage in healthcare and self-care. Training to promote non-discriminatory behaviours towards people who may struggle with literacy tasks would also be welcome. Even short basic interventions might serve to encourage simplified communication and a reduction in the use of ‘on the spot’ literacy activities.

The findings of our study caution quite strongly against the use of formal literacy screening, particularly outside the context of an already established and trusted patient-clinician relationship. They reinforce previously expressed concerns about its potential to cause harm in the form of significant anxiety, shame and alienation [41,42]. Although some patients have been willing to express some support for literacy screening [43] this was not unequivocal [44] and the risk of exacerbating disengagement from healthcare contact compounds concerns that the practice is unlikely to be cost or time effective. Participants in the present study most prominently recommended universal solutions to their problems (simplifying the presentation of information and offering non-stigmatising alternatives to self-completion of literacy tasks to everyone, not just those with low literacy). Universal solutions have been advocated by others in the interests of improving health literacy in the general population, not only among those with low literacy [38]. Techniques such as Teach-back, which helps healthcare providers to confirm that they have explained what the patient needs to know in a way that the patient understands have been developed and tested in the USA [36] and would benefit from wider implementation.

### Implications for further research

Most health literacy research, including the testing and evaluation of initiatives to improve health literacy or improve outcomes for those with low literacy, has been carried out in the US. The findings of this study have highlighted particular issues through first-hand accounts of people with low literacy with reference to the UK health system. While some suggestions for service improvements need not be tested, for example, the colour coding within the hospital environment, or awareness raising among healthcare staff, others would benefit from piloting and robust evaluation. These could include the use of Teach-back and similar techniques in clinical departments. As well as evaluating outcomes, research should focus on the acceptability of interventions to people with low and those with higher literacy levels.

## Conclusion

When health services and health professionals adopt practices that assume significant literacy skills, their effectiveness for a large but often hidden population of people with low literacy is limited. This study has shown that the stigma associated with low literacy, as well as the consequences of literacy-related mistakes, can impair people’s spoken engagement with healthcare staff, and potential to benefit from health services. Unless problems with *both* written information and interpersonal communication are acknowledged and addressed, policy ambitions to improve the experiences, effectiveness and safety of healthcare, and to reduce social inequalities in health are unlikely to be realised.

## Competing interests

The authors declare that they have no competing interests.

## Authors’ contributions

PE formed the concept of the study, led on the study and topic guide design, carried out all interviews and focus groups, led on the analysis of the data and wrote the first draft of the article. VAE participated in the study and topic guide design, contributed to the analysis of the data and critically reviewed and revised the manuscript. BW participated in the study and topic guide design, contributed to the analysis of the data and contributed to the review and revision of the manuscript. All authors read and approved the final manuscript.

## Authors’ information

VAE and BW were both formerly at the Social Dimensions of Health Institute, Universities of Dundee and St Andrews.

## Pre-publication history

The pre-publication history for this paper can be accessed here:

http://www.biomedcentral.com/1472-6963/13/319/prepub

## References

[B1] Moser CS Improving literacy and numeracy: a fresh start 1999 London: DfEE Publications

[B2] Mayor S Nearly half of adults in England don’t understand health information material, study indicates BMJ 2012 345 e8364 10.1136/bmj.e8364 23220559

[B3] Sorensen K Van den Broucke S Fullam J Doyle G Pelikan J Slonska Z Brand H (HLS-EU) Consortium Health Literacy Project European Health literacy and public health: a systematic review and integration of definitions and models BMC Publ Health 2012 12 80 10.1186/1471-2458-12-80 PMC3292515 22276600

[B4] Zarcadoolas C Greer DS Pleasant A Understanding health literacy: an expanded model Health Promot Internation 2005 20 2 195 203 10.1093/heapro/dah609 15788526

[B5] Ishikawa H Yano E Patient health literacy and participation in the health-care process Health Expect 2008 11 2 113 122 10.1111/j.1369-7625.2008.00497.x 18494956 PMC5060442

[B6] Dewalt DA Berkman ND Sheridan S Lohr KN Pignone MP Literacy and health outcomes: a systematic review of the literature J Gen Intern Med 2004 19 12 1228 1239 10.1111/j.1525-1497.2004.40153.x 15610334 PMC1492599

[B7] Baker DW Parker RM Williams MV Clark WS Nurss J The relationship of patient reading ability to self-reported health and use of health services Am J Public Health 1997 87 6 1027 1030 10.2105/AJPH.87.6.1027 9224190 PMC1380944

[B8] Baker DW Parker RM Williams MV Clark WS Health literacy and the risk of hospital admission J Gen Intern Med 1998 13 12 791 798 10.1046/j.1525-1497.1998.00242.x 9844076 PMC1497036

[B9] Kalichman SC Rompa D Functional health literacy is associated with health status and health-related knowledge in people living with HIV-AIDS JAIDS 2000 25 4 337 344 10.1097/00126334-200012010-00007 11114834

[B10] Berkman ND Sheridan SL Donahue KE Halpern DJ Crotty K Low health literacy and health outcomes: an updated systematic review Ann Intern Med 2011 155 2 97 107 10.7326/0003-4819-155-2-201107190-00005 21768583

[B11] Williams MV Baker DW Honig EG Lee TM Nowlan A Inadequate literacy is a barrier to asthma knowledge and self-care Chest 1998 114 4 1008 1015 10.1378/chest.114.4.1008 9792569

[B12] Mancuso CA Rincon M Impact of health literacy on longitudinal asthma outcomes J Gen Intern Med 2006 21 8 813 817 10.1111/j.1525-1497.2006.00528.x 16881939 PMC1831574

[B13] Williams MV Baker DW Parker RM Nurss JR Relationship of functional health literacy to patients’ knowledge of their chronic disease. A study of patients with hypertension and diabetes Arch Intern Med 1998 158 2 166 172 10.1001/archinte.158.2.166 9448555

[B14] Schillinger D Grumbach K Piette J Wang F Osmond D Daher C Palacios J Sullivan GD Bindman AB Association of health literacy with diabetes outcomes JAMA 2002 288 4 475 482 10.1001/jama.288.4.475 12132978

[B15] Bynner J Parsons S New light on literacy and numeracy 2006 London: National Research and Development Centre for Adult Literacy and Numeracy

[B16] Bostock S Steptoe A Association between low functional health literacy and mortality in older adults: longitudinal cohort study BMJ 2012 344 e1602 10.1136/bmj.e1602 22422872 PMC3307807

[B17] Organisation for Economic Co-operation and Development Literacy, economy and society: Results of the first international literacy survey 1995 Paris: OECD

[B18] Parikh NS Parker RM Nurss JR Baker DW Williams MV Shame and health literacy: the unspoken connection Patient Educ Couns 1996 27 1 33 39 10.1016/0738-3991(95)00787-3 8788747

[B19] Bass PF Wilson JF 3rd Griffith CH Barnett DR Residents’ ability to identify patients with poor literacy skills Acad Med 2002 77 10 1039 1041 10.1097/00001888-200210000-00021 12377684

[B20] Marcus EN The silent epidemic–the health effects of illiteracy N Engl J Med 2006 355 4 339 341 10.1056/NEJMp058328 16870912

[B21] Safeer RS Keenan J Health literacy: the gap between physicians and patients Am Fam Physician 2005 72 3 463 468 16100861

[B22] Easton P Entwistle VA Williams B Health in the ‘hidden population’ of people with low literacy. A systematic review of the literature BMC Publ Health 2010 10 459 10.1186/1471-2458-10-459 PMC2923110 20687946

[B23] Paasche-Orlow MK Wolf MS The causal pathways linking health literacy to health outcomes Am J Health Behav 2007 31 Suppl 1 S19 S26 17931132 10.5555/ajhb.2007.31.supp.S19

[B24] Department of Health Equity and excellence: liberating the NHS 2010 UK: The Stationery Office

[B25] The Scottish Government Better health, better care: action plan 2007 Edinburgh: The Scottish Government

[B26] The Scottish Government The healthcare quality strategy for NHS Scotland 2010 Edinburgh: The Scottish Government

[B27] The Scottish Index of Multiple Deprivation http://www.scotland.gov.uk/Topics/Statistics/SIMD Accessed 11/06/2013

[B28] General Register Office for Scotland http://www.gro-scotland.gov.uk/ Accessed 11/06/2013

[B29] Ritchie J Lewis J Qualitative research practice. A guide for social science students and researchers 2009 London: Sage Publications Ltd

[B30] Williams MV Parker RM Baker DW Parikh NS Pitkin K Coates WC Nurss JR Inadequate functional health literacy among patients at two public hospitals JAMA 1995 274 21 1677 1682 10.1001/jama.1995.03530210031026 7474271

[B31] Clement WA Wales Y Readability and content of postoperative tonsillectomy instructions given to patients in Scotland Clin Otolaryngol Allied Sci 2004 29 2 149 152 10.1111/j.0307-7772.2004.00757.x 15113300

[B32] Kutner M Greenberg E Jin Y Paulsen C White S The health literacy of America’s adults: results from the 2003 National Assessment of Adult Literacy. NCES 2006–483 2006 Washington DC: US Department of Education National Center for Education Statistics

[B33] Davis TC Wolf MS Bass PF Middlebrooks M 3rd Kennen E Baker DW Bennett CL Durazo-Arvizu R Bocchini A Savory S Parker RM Low literacy impairs comprehension of prescription drug warning labels J Gen Intern Med 2006 21 8 847 851 10.1111/j.1525-1497.2006.00529.x 16881945 PMC1831578

[B34] Easton P Entwistle V Williams B Exploring the links between low literacy and poor health. An investigation of healthcare and self-care experiences 2010 Edinburgh: Chief Scientist Office for Scotland

[B35] Easton PM Exploring the pathways to poor health in the ‘hidden population’ with low literacy 2011 PhD Thesis. Dundee: University of Dundee http://discovery.dundee.ac.uk/portal/files/1199257/Easton_phd_2011.pdf

[B36] DeWalt DA Callahan LF Hawk VH Broucksou KA Hink A Health literacy universal precautions toolkit AHRQ: http://www.ahrq.gov/legacy/qual/literacy/ Accessed 11/06/2013 10.1016/j.outlook.2010.12.002 PMC5091930 21402204

[B37] Entwistle V Firnigl D Ryan M Francis J Kinghorn P Which experiences of health care delivery matter to service users and why? A critical interpretive synthesis and conceptual map J Health Serv Res Policy 2012 17 70 78 10.1258/jhsrp.2011.011029 21967821 PMC3336938

[B38] Raynor DK Health literacy BMJ 2012 344 e2188 10.1136/bmj.e2188 22442354

[B39] The Scottish Government Equally well: report of the ministerial task force on health inequalities 2008 Edinburgh: The Scottish Government

[B40] Entwistle V Cribb A Enabling people to live well: fresh thinking about collaborative approaches to care for people with long-term conditions 2013 London: The Health Foundation

[B41] Paasche-Orlow MK Wolf MS Evidence does not support clinical screening of literacy J Gen Intern Med 2007 23 1 100 102 17992564 10.1007/s11606-007-0447-2 PMC2173929

[B42] Wolf MS Williams MV Parker RM Parikh NS Nowlan AW Baker DW Patients’ shame and attitudes toward discussing the results of literacy screening J Health Commun 2007 12 721 732 10.1080/10810730701672173 18030638

[B43] VanGeest JB Welch VL Weiner SJ Patients’ Perceptions of screening for health literacy: reactions to the newest vital sign J Health Commun 2010 15 4 402 412 10.1080/10810731003753117 20574878

[B44] Hahn EA Garcia SF Du H Cella D Patient attitudes and preferences regarding literacy screening in ambulatory cancer care clinics Patient Relat Outcome Meas 2010 1 19 27 22915949 10.2147/prom.s9361 PMC3417894

